# Association of occlusal support with type 2 diabetes: A community-based study

**DOI:** 10.3389/fendo.2022.934274

**Published:** 2022-08-08

**Authors:** Dongxin Da, Suyu Ge, Hao Zhang, Xiaoli Zeng, Yiwei Jiang, Jin Yu, Huning Wang, Wanqing Wu, Zhenxu Xiao, Xiaoniu Liang, Ding Ding, Ying Zhang

**Affiliations:** ^1^ Department of Preventive Dentistry, Shanghai Stomatological Hospital & School of Stomatology, Fudan University, Shanghai, China; ^2^ Shanghai Key Laboratory of Craniomaxillofacial Development and Diseases, Fudan University, Shanghai, China; ^3^ Institute of Neurology, Huashan Hospital, Fudan University, Shanghai, China; ^4^ National Center for Neurological Disorders, Huashan Hospital, Fudan University, Shanghai, China; ^5^ National Clinical Research Center for Aging and Medicine, Huashan Hospital, Fudan University, Shanghai, China

**Keywords:** diabetes, occlusion, mastication, nutrition, dental health survey

## Abstract

Occlusal support was proved to be associated with type 2 diabetes. Our aim was to investigate the association between the Eichner index and the prevalence of type 2 diabetes. We included 715 participants with oral health examinations in the Shanghai Aging Study. The occlusal support status was determined by the number of functional occlusal support areas and Eichner index classifications. Those with fasting plasma glucose ≥126 mg/dL and/or hemoglobin A1c ≥6.5% and/or current medications for type 2 diabetes with relevant medical history were diagnosed with type 2 diabetes. Multiple logistic regression models were used to analyze the relationship between occlusal support status and type 2 diabetes. The average age of 715 participants was 73.74 ± 6.49 years old. There were 84 diabetics with 1.71 occlusal supporting areas on average. Seven hundred and fifteen participants were divided into 3 groups according to Eichner classifications: Eichner group A with 4 occlusal functional areas, Eichner group B with 1-3 occlusal functional areas or 0 area with anterior occlusal contact, and Eichner group C with no functional occlusal contact. Blood glucose level was significantly lower in participants of Eichner group A compared to those in group B or C. The ordinal logistic regression showed more occlusal supporting areas were significantly associated with less type 2 diabetes cases with an Odds Ratio(OR) of 0.253(95%CI 0.108-0.594) after adjusting covariates. Participants in Eichner group A had a significantly much lower OR of 0.078 for type 2 diabetes (95%CI 0.009-0.694) compared to those in Eichner group C after adjustment. The number of functional occlusal support areas might be inversely related to the blood glucose level and the prevalence of type 2 diabetes.

## Introduction

Type 2 diabetes is one of the world’s commonest chronic diseases. Itis a group of metabolic disorders characterized by hyperglycemia caused by the insulin insufficiency, pancreas dysfunction and augmentation of insulin resistance (1). Excluding COVID-19 related deaths, an estimated 6.7 million adults died from diabetes or its complications in 2021, accounting for 12.2% of all-cause mortality globally (2). In China, the prevalence of diabetes was approximately 10% in 2021 and expected to reach 15% by 2045 (3).

Metabolic disorders of blood glucose, lipid and protein caused by type 2 diabetes could lead to systemic chronic inflammation including periodontitis (4). Periodontitis is a chronic infectious and inflammatory disease which occurred in oral soft tissues like gingiva and hard tissues like alveolar bone and caused by dental plaque (5). Type 2 diabetes might reduce the immune response and self-restoration capacity of oral tissues, making periodontitis worse, teeth loosening and occlusal support declining (6). Coelho et al. found significant association between dental caries and type 1 diabetes (7–10). There was also significant association between dental caries and tooth loss,which was elucidated by Ana Sofia Coelho et al. (11).Tooth loss and declining occlusal support could significantly reduce mastication, thus affecting nutrients absorption and indirectly aggravating gastrointestinal burden (12).

Occlusal support is crucial for mastication and aesthetics. Studies had shown that patients without occlusal support of natural molars had significantly reduced masticatory function compared with those with incomplete arches (13–15). Kosaka et al. found that the number of functional teeth and occlusal support areas were significantly correlated with masticatory function in Japanese elderly population (14). Significant associations were also found between functional occlusal support areas and masticatory performance due to the number and location of remaining teeth (15, 16). As the most widely used classification of occlusal support (15), Eichner index has been used to evaluate the occlusal function of patients with incomplete and edentulous dentition according to functional occlusal support areas (13, 14, 17).

There were multiple studies on the association between occlusal support and type 2 diabetes (18–21). More evidence was needed to further investigate the association between occlusal support and type 2 diabetes. Our study aimed to elucidate the association between the Eichner index or number of occlusal support areas and type 2 diabetes among older Chinese community-dwelling adults. We also compared the differences in nutritional intake between the different Eichner groups to control the effect of nutritional intake on type 2 diabetes. We aimed to investigate the association between occlusal support and type 2 diabetes and the null hypothesis was that there was no association between type 2 diabetes and occlusal support.

## Materials and methods

### Study population

The Shanghai Aging Study recruited participants from a community of Jing’an District in downtown Shanghai, China. Detailed recruitment procedure was of the published elsewhere (22). Inclusion criteria of the current study included those (1) on the list of permanent residents; (2) aged ≥60 years; (3) were administered the oral health examination; (4) accepted blood glucose measurement and type 2 diabetes assessment. Potential participants were excluded if they: (1) were deceased; (2) had severe problems of vision, hearing, or speaking, and were not able to participate actively in the face-to-face survey clinical evaluation; or (3) refused to participant the study.

The Medical Ethics Committee of Huashan Hospital, Fudan University approved the study (No. HIRB2009-195). Written informed consent was obtained from all participants and/or their legally acceptable representatives.

Our study was designed and conducted according to the STROBE (STrengthening the Reporting of OBservational studies in Epidemiology) guidelines (**Supplementary STROBE checklist**).

### Oral health examination

One dentist conducted the oral health examination for all participants. Occlusal support was evaluated by Eichner index, which described the functional occlusal areas of premolars and molars through oral examination. There were four occlusal supporting areas including left premolars, right premolars, left molars and right molars. Antagonistic occlusal contacts by natural teeth, crowns, or fixed partial dentures were recorded (23). The participants were divided into 3 groups: Eichner group A with 4 occlusal functional areas, Eichner group B with 1-3 occlusal functional areas or 0 area with anterior occlusal contact, and Eichner group C with no functional occlusal contact.

### Blood glucose measurement and diabetes diagnosis

Blood glucose was measured from the serum from participants’ venous blood in the central laboratory in Huashan hospital. Those with fasting plasma glucose ≥126 mg/dL or 2-hour plasma glucose ≥200mg/dl and/or current medications for type 2 diabetes with relevant medical history were diagnosed with type 2 diabetes.

### Data collection of other variables

Trained doctors and nurses made face-to-face interviews with participants, asking them for basic demographic information (age, sex, height, weight, etc.), lifestyle habits (smoking, alcohol drinking), social information (years of education), and chronic disease (type 2 diabetes, hypertension). BMI is calculated by dividing a person’s weight(kilograms) by his height(meters) squared. Self-reported chronic diseases were checked against medical records before registration. Detailed definitions of the above variables have been previously published (24).

Trained investigators interviewed the participants face-to-face on the daily dietary intake including amount and frequency over the past 12 months by a 111-item interviewer-administered food frequency questionnaire (FFQ), which has been previously validated in Chinese population (25). Intakes of total macronutrients and micronutrients were calculated based on data from the FFQ and the “China Food Composition, 2nd Edition” (26). These nutrients included protein, carbohydrates, total and saturated fat, cholesterol, fruits and vegetables, sugar, and a range of micronutrients (vitamins A, C and E, iron, β-carotene, and vitamin K1).

### Statistical analysis

In the current study, rates of participants with Eichner classification B and C in type 2 diabetes group and non-diabetes group were 0.833 and 0.702, due to the sample size of 715, the power of test (1−β) exceeded 95% with α=0.05, according to the sample size calculation formula, The sample size was enough in our study.

Data were analyzed using SAS 9.4 (SAS Institute Inc., Cary, NC, USA). Continuous variables were described in mean and standard deviation (SD), and frequencies (%) were used for categorical variables. We used the Student t-test, Pearson Chi-squared test, analysis of variance (ANOVA) to compare the variables. The association between the number of occlusal supporting areas/Eichner index and type 2 diabetes was examined by logistic regression model. The number of occlusal supporting areas was treated as a continuous variable while the Eichner index as an ordinal categorical variable. The Odds ratio (OR) and 95% confidence intervals (CI) were presented as the measurement of the association. Model 1 was a univariate model and Model 2 and 3 were multivariable models. Model 2 was adjusted for age and sex; Model 3 was adjusted for confounders such as age, sex, body mass index, education, smoking and alcohol drinking, blood glucose and hypertension. In the model assessing the association between Eicher index and diabetes, group C was the reference group.

The p-values and 95% CIs were estimated in a two-tailed manner, and p<0.05 was considered significant.

## Results

From March 2016 to December 2019, a total of 715 among 1064 participants receiving oral health examination, blood glucose measurement and type 2 diabetes assessment in the Shanghai Aging Study were included in the current study. Among the rest of 349 individuals, 30 did not undergo the blood glucose/type 2 diabetes assessment, and 319 refused the oral health examination. The average age was 73.74 ± 6.49 years old. There were 84 diabetics with 1.71 occlusal supporting areas on average. Significant difference in sex, age, cigarette smoking, alcohol drinking, prevalence of type 2 diabetes, blood glucose concentration was found between patients with different Eichner classifications. Participants in Eichner group A had a significantly lower prevalence of type 2 diabetes than those in group B or C(P<0.05). Blood glucose level was significantly lower in Eichner group A compared to group B or C (**Table 1**). The Eichner classification index was not associated with the daily nutrition intake including total calorie, protein, fat, carbohydrate (**Table 1**).

**Table 1:** Demographic, life style, and medical history of the participants with different Eichner classifications.

	ALL (N=715)	Group A (N=202)	Group B (N=378)	Group C (N=135)	P value
Sex					0.0004
Female, n(%)	386 (53.99)	131 (18.32)	195 (27.27)	60 (8.39)	
Male, n(%)	329 (46.01)	71 (9.93)	183 (25.59)	75 (10.49)	
Age,years, mean ± SD	73.74 ± 6.49	71.14 ± 5.67	73.95 ± 6.18	77.05 ± 6.89	<.0001
Education years,mean ± SD	11.99 ± 3.49	12 ± 3.29	12.12 ± 3.41	11.6 ± 3.99	0.3297
Body mass index,mean ± SD	24.55 ± 3.42	24.31 ± 3.47	24.59 ± 3.43	24.81 ± 3.31	0.4025
Cigarette smoking, n(%)	91(12.73)	91(12.73)	15 (2.10)	42 (5.87)	<.0001
Alcohol drinking, n (%)	66 (9.23)	10 (1.40)	36 (5.03)	20 (2.80)	0.0087
Hypertension, n(%)	326 (45.59)	88 (12.31)	180(25.17)	58 (8.11)	0.5125
type 2 diabetes, n (%)	84 (11.75)	14 (1.96)	56 (7.83)	14 (1.96)	0.0166
Occlusal supporting areas,mean ± SD	2.01 ± 1.62	4.00 ± 0.00	1.66 ± 1.14	0 ± 0	<.0001
Blood glucose, mmol/L, mean ± SD	5.67 ± 1.51	5.49 ± 0.85	5.68 ± 1.62	5.91 ± 1.88	0.0389
Calorie, kcal, mean ± SD	1284.12 ± 428.96	1298.36 ± 428.46	1271.46 ± 432.43	1288.28 ± 426.43	0.7978
Protein, g, mean ± SD	72.70 ± 30.79	74.34 ± 30.54	71.45 ± 28.80	72.91 ± 33.50	0.6191
Fat, g, mean ± SD	41.20 ± 20.47	42.01 ± 19.12	40.42 ± 20.66	41.50 ± 21.41	0.7008
Carbohydrate, g, mean ± SD	189.71 ± 60.10	190.06 ± 59.96	189.12 ± 60.95	190.18 ± 59.41	0.9780

Group A:4 occlusal functional areas; Group B: 1-3 occlusal functional areas or 0 area with anterior occlusal contact; Group C: with no functional occlusal contact. Continuous variables including age, education years, body mass index, occlusal supporting areas, blood glucose, calories, protein, fat, carbohydrate were described in mean and standard deviation (SD), and frequencies (%) were used for categorical variables including cigarette smoking, alcohol drinking, hypertension, type 2 diabetes. Analysis of variance (ANOVA) was used to compare the continuous variables and Pearson Chi-squared test to compare the categorical variables.

**Figure 1:**
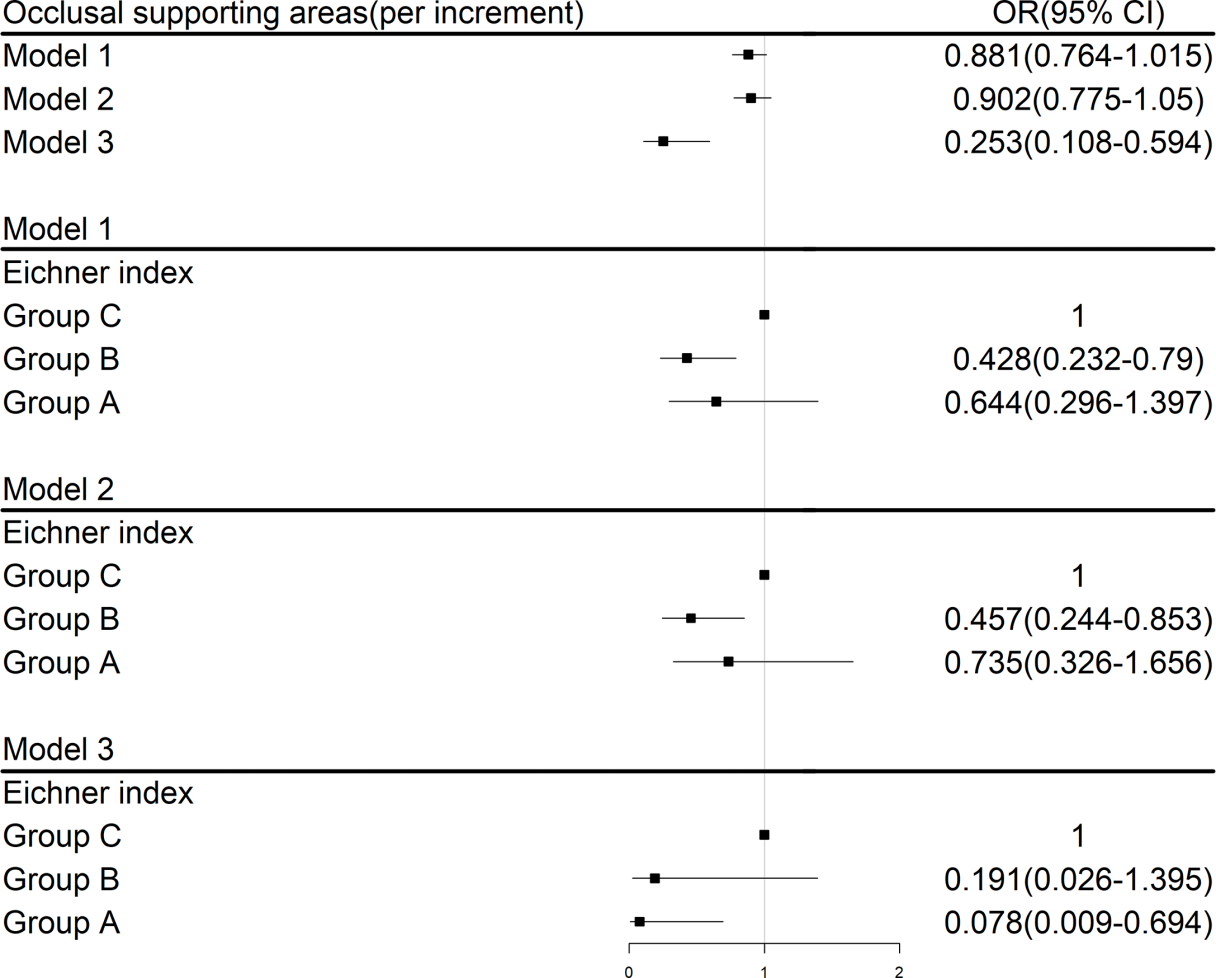
Logistic regression analysis for risk factors associated with type 2 diabetes; Group A:4 occlusal functional areas; Group B: 1-3 occlusal functional areas or 0 area with anterior occlusal contact; Group C: with no functional occlusal contact; Model 1:univariate; Model 2: adjusted for sex and age; Model 3: adjusted for age, sex, body mass index, education years, smoking and alcohol drinking, blood glucose, hypertension.

The ordinal logistic regression showed more occlusal supporting areas were significantly associated with lower risk of type 2 diabetes with an OR of 0.253(95%CI 0.108-0.594). Participants with Eichner group B had lower risk of suffering from diabetes with an OR of 0.457 (95%CI 0.244-0.853) in model 2 compared to Eichner group C. After adjusting for sex, age, BMI, education years, hypertension and blood glucose, participants with Eichner group A had a significantly lower OR for type 2 diabetes of 0.078 (95%CI 0.009-0.694) compared to group A after adjustment (**Figure 1**).

## Discussion

In the cross-sectional study, the null hypothesis was there was no association between occlusal support and type 2 diabetes. We used functional occlusal support areas and Eicher index to evaluate the occlusal support status. There was significant association between lower occlusal support and higher prevalence of type 2 diabetes.

Masticatory performance could be evaluated by several indicators including tooth loss, number of occlusal support areas and Eichner index (27, 28). There was closely bidirectional relationship between tooth loss and worse occlusal support. The cross-sectional and longitudinal study in Suita found significant relationship between tooth loss and lower occlusal support areas (14). Fushida found decreased posterior occlusal support areas accelerated tooth loss in the general Japanese urban population (29). Eichner index was found to be correlated with tooth loss by kebe (13). However, compared to tooth loss, number of occlusal support areas and Eichner index could explain 10%-20% of the variation in bite force and masticatory performance (27). So we selected them as the indicators of chewing function.

Older adults with worse occlusal support had higher risk of type 2 diabetes, which was not elucidated in previous studies (18, 30). However, risk of type 2 diabetes was proved to be significantly associated with tooth loss (18, 31), which was closely correlated with lower occlusal support (17, 32). Demmer et al. found missing ≥ 25 teeth was significantly associated with increased incidence of diabetes compared to those missing 0-8 teeth (33). A longitudinal study in Finland showed missing ≥9 teeth was significantly associated with higher incidence of diabetes (20). Fushida et al. found lower masticatory performance a risk factor for the development of high fasting plasma glucose after surveying 599 participants in the Suita study at baseline and follow-up (34). Possible mechanisms explaining the correlation between declining occlusal support and type 2 diabetes might be due to ongoing chronic inflammation, repeated periodontium-originated bacteremia and masticatory dysfunction (20). Inflammatory cytokines and lipopolysaccharide from periodontal bacteria might spread systemically to cause metabolic disorders like diabetes (35–37). The declining masticatory function due to tooth loss and decreasing occlusal support might cause the change of nutrients absorption, thus increasing the risk of type 2 diabetes (38).

However, there was not significant association between the nutrients intake and different Eichner index classifications, which is inconsistent with some studies (39–41). We found participants with Eichner index A had lower blood glucose level with equal nutrients intake. Although thorough mastication could raise plasma glucose levels by thoroughly grinding the food and mixing it adequately with enzymes and stomach acids (42), it can also raise insulin secretion and ultimately lower blood glucose levels (21). This speculation was proved by Suzuki et al. who found compared with poor mastication, good mastication might reduce postprandial blood glucose concentration by improving the digestion and absorption of nutrients in Japanese population. Thorough mastication raised postprandial blood glucose after 30 minutes, but also significantly raised insulin levels, ultimately leading to lower postprandial blood glucose after 2 hours (21). However, such effect of thorough mastication on glucose levels by stimulating insulin secretion still needed more evidence.

Our study had several advantages. It was the first Chinese epidemiological study to investigate the association between occlusal support and type 2 diabetes in community-dwelling older adults to our knowledge. We adjusted relevant covariates sequentially in three models. However, our study had several limitations. Firstly, the sample size might be relatively small. The odds ratio decreased sharply due to the sudden decrease in occlusal support in Model 3 due to the relatively small sample size and many covariates. However, the sample size was proved to be enough after power analysis. Secondly, we did not measure risk factors of type 2 diabetes including the maximum bite force, periodontal conditions and denture fitting. As an indicator of masticatory performance, maximum bite force may also increase the risk of type 2 diabetes (20, 21, 38). However, the maximum bite force was closely related to functional occlusal area and Eichner index (43), and the effects of the latter two were analyzed respectively by using a multivariable logistic regression model to control confounding factors. Thirdly, the intake nutrients were so limited that some underlying nutrients might be neglected which caused metabolic disorders. Fourthly, occlusal support was both a result and cause of various oral health factors. As occlusal support failed to represent all oral health factors, more research was needed to clarify the association between oral health and type 2 diabetes. Finally, the cross-sectional study was unable to reveal the causal relationship between occlusal support and type 2 diabetes. The specific mechanism of thorough mastication on blood glucose remained unknown. Further studies were essential to reveal the causal relationship between occlusal support, tooth loss, digestive function, plasma glucose and type 2 diabetes.

## Conclusions

In conclusion, less occlusal functional areas, leading to worse occlusal support, might be related to higher blood glucose concentration and prevalence of type 2 diabetes. Our study suggested that older adults should actively prevent tooth loss to reduce the risk of type 2 diabetes. More studies on their association needed to be carried out.

## Data availability statement

The raw data supporting the conclusions of this article will be made available by the authors, without undue reservation.

## Ethics statement

The studies involving human participants were reviewed and approved by The Medical Ethics Committee of Huashan Hospital, Fudan University. The patients/participants provided their written informed consent to participate in this study.

## Author contributions

DDa: Contributed to conception, design, data acquisition and interpretation, drafted and critically revised the manuscript. SG: Contributed to data acquisition and interpretation. HZ: Contributed to statistical analysis. XZ: Contributed to data acquisition and interpretation. JY: Contributed to data acquisition and interpretation. YJ: Contributed to data acquisition and interpretation. HW: Contributed to data acquisition and interpretation. WW: Contributed to data acquisition and interpretation. ZX: Contributed to data acquisition and interpretation. XL: Contributed to data acquisition and interpretation. DDi: Contributed to conception, design, and critically revised the manuscript. YZ: Contributed to conception, design, and critically revised the manuscript. All authors contributed to the article and approved the submitted version.

## Funding

Shanghai Municipal Science and Technology Major Project [2018SHZDZX01] and ZJ LAB, Key Project of the Ministry of Science and Technology, China [2021YFE0111800] supported data and sample collection. The Shanghai Stomatological Hospital School-level Key Department and Innovative Team Project [grant number SSDC-2019-ZDXK01, SSDC-2020-CXTD-A03] and Clinical Research Program from Shanghai Health Commission[grant number 2020YJZX0117, 20194Y0142] supported analysis, interpretation of data and writing the manuscript.

## Acknowledgments

The authors thank all the study participants for their cooperation, and Ms. Fang Pei, Zhaolan Ding, Yiping Wang for their efforts of study coordination.

## Conflict of interest

The authors declare that the research was conducted in the absence of any commercial or financial relationships that could be construed as a potential conflict of interest.

## Publisher’s note

All claims expressed in this article are solely those of the authors and do not necessarily represent those of their affiliated organizations, or those of the publisher, the editors and the reviewers. Any product that may be evaluated in this article, or claim that may be made by its manufacturer, is not guaranteed or endorsed by the publisher.
