# Supplementary material for: Association of occlusal support with type 2 diabetes: A community-based study
Source: Front Endocrinol (Lausanne). 2022 Aug 8;13:934274. doi: 10.3389/fendo.2022.934274 (PMC9393363; doi:10.3389/fendo.2022.934274)
Supplement: Supplementary file 1 [file Table_1.doc]

STROBE Statement—Checklist of items that should be included in reports of ***case-control studies***

|  | Item No | Recommendation |
| --- | --- | --- |
| **Title and abstract** | 1 | (*a*) Indicate the study’s design with a commonly used term in the title or the abstract  A Community-Based Study |
|  |  | (*b*) Provide in the abstract an informative and balanced summary of what was done and what was found  Oral health examinations and blood glucose measurements were conducted on the community-dwelling adults. The number of functional occlusal support areas may be inversely related to the blood glucose level and the prevalence of DM. |
| Introduction | | |
| Background/rationale | 2 | Explain the scientific background and rationale for the investigation being reported  Diabetes mellitus (DM) is one of the world’s commonest chronic diseases. It is a group of metabolic disorders characterized by hyperglycemia caused by the insufficiency of insulin and/or the dysfunction of the pancreas(2021). Excluding COVID-19 related deaths, an estimated 6.7 million adults died from diabetes or its complications in 2021, accounting for 12.2% of all-cause mortality globally(Sapra and Bhandari 2021). In China, the prevalence of diabetes was approximately 10% in 2021 and expected to reach 15% by 2045(Cheng et al. 2021).  Metabolic disorders of blood glucose, lipid and protein caused by DM could lead to systemic chronic inflammation including periodontitis(Ramos-Garcia et al. 2021). Periodontitis is a chronic infectious and inflammatory disease which occurred in oral soft tissues like gingiva and hard tissues like alveolar bone and caused by dental plaque(Mehrotra and Singh 2021). DM might reduce the immune response and self-restoration capacity of oral tissues, making periodontitis worse, teeth loosening and occlusal support declining(Luong et al. 2021). Tooth loss and declining occlusal support could significantly reduce mastication, thus affecting nutrients absorption and indirectly aggravating gastrointestinal burden(Lee et al. 2021).  Occlusal support is crucial for mastication and aesthetics. Studies had shown that patients without occlusal support of natural molars had significantly reduced masticatory function compared with those with incomplete arches(Ikebe et al. 2010; Kosaka et al. 2016; Tanaka and Shiga 2018). KosakaT et al. found that the number of functional teeth and occlusal support areas were significantly correlated with masticatory function in Japanese elderly population(Kosaka et al. 2016).Significant associations were also found between functional occlusal support areas and masticatory performance due to the number and location of remaining teeth(Tanaka and Shiga 2018; Kinoshita et al. 2021).As the most widely used classification of occlusal support(Tanaka and Shiga 2018), Eichner index has been used to evaluate the occlusal function of patients with incomplete and edentulous dentition according to functional occlusal support areas(Ikebe et al. 2010; Kosaka et al. 2016; Kosaka et al. 2018).  There were multiple studies on the association between occlusal support and DM. More evidence was needed to further investigate the association between occlusal support and DM. |
| Objectives | 3 | State specific objectives, including any prespecified hypotheses  Our study aimed to elucidate the association between the Eichner index or number of occlusal support areas and DM among older Chinese community-dwellings. We also compared the differences in nutritional intake between the different Eichner groups to control the effect of nutritional intake on DM. We hypothesized worse occlusal support could be associated with higher prevalence of DM. |
| Methods | | |
| Study design | 4 | Present key elements of study design early in the paper  Case-control study |
| Setting | 5 | Describe the setting, locations, and relevant dates, including periods of recruitment, exposure, follow-up, and data collection  From March 2016 to December 2019, 715 participants aged ≥ 60 years were enrolled in Jing’an District from the Shanghai Aging Study. The recruitment procedure for the Shanghai Aging Study had been published elsewhere(Ding et al. 2014). |
| Participants | 6 | (*a*) Give the eligibility criteria, and the sources and methods of case ascertainment and control selection. Give the rationale for the choice of cases and controls  Inclusion criteria included being on the list of permanent residents and being ≥60 years old. Potential participants were excluded if they: (1) were deceased; (2) showed severe schizophrenia or mental retardation on their medical record; or (3) had severe  problems of vision, hearing, or speaking, and were not able to participate actively in the neuropsychological evaluation. Cases were those with diabetes, controls were those without diabetes. |
|  |  | (*b*) For matched studies, give matching criteria and the number of controls per case  Not applicable. |
| Variables | 7 | Clearly define all outcomes, exposures, predictors, potential confounders, and effect modifiers. Give diagnostic criteria, if applicable  Outcomes: suffered from diabetes or not  Exposures: different number of occlusal support areas and Eichner index  Potential confounders: age, sex, body mass index, education, smoking and alcohol drinking, blood glucose, hypertension and the interaction between glucose and number of occlusal support areas and Eichner index classification.  Effect modifiers: not applicable.  Diagnostic criteria: The occlusal support status was determined by the number of functional occlusal support areas and Eichner index classifications. Those with fasting plasma glucose ≥126 mg/dL and/or haemoglobin A1c ≥6.1% and/or current medications for DM with relevant medical history were diagnosed with Diabetes. |
| Data sources/ measurement | 8* | For each variable of interest, give sources of data and details of methods of assessment (measurement). Describe comparability of assessment methods if there is more than one group  One dentist conducted the oral health examination for all participants. Occlusal support was evaluated by Eichner index, which described the functional occlusal areas of premolars and molars through oral examination. There were four occlusal supporting areas including left premolars, right premolars, left molars and right molars. Antagonistic occlusal contacts by natural teeth, crowns, or fixed partial dentures were recorded(Eichner 1990). The participants were divided into 3 groups: Eichner group A with 4 occlusal functional areas, Eichner group B with 1-3 occlusal functional areas or 0 area with anterior occlusal contact, and Eichner group C with no functional occlusal contact.  Blood glucose was measured from the serum from participants’ venous blood in the central laboratory in Huashan hospital. Those with fasting plasma glucose ≥126 mg/dL and/or haemoglobinA1c ≥6.1% and/or current medications for DM with relevant medical history were diagnosed with DM.  Data collection of other variables  Trained doctors and nurses made face-to-face interviews with participants, asking them for basic demographic information (age, sex, height, weight, etc.), lifestyle habits (smoking, alcohol drinking), social information (years of education), and chronic disease (DM, hypertension). BMI is calculated by dividing a person's weight (kilograms) by his height (meters) squared. Self-reported chronic diseases were checked against medical records before registration. Detailed definitions of the above variables have been previously published(Ding et al. 2021).  Trained investigators interviewed face-to-face with the participants on the daily intake and frequency of all food and beverages. Intakes of total macronutrients and micronutrients were calculated using a validated computer program based on the China Food Composition(Yang et al. 2009). These nutrients included protein, carbohydrates, total and saturated fat, cholesterol, fruits and vegetables, sugar, and a range of micronutrients (vitamins A, C and E, iron, β-carotene, and vitamin K1). Total calories, protein, cholesterol, and carbohydrate intake were calculated based on these intakes. |
| Bias | 9 | Describe any efforts to address potential sources of bias |
| Study size | 10 | Explain how the study size was arrived at  In the current study, rates of participants with Eichner classification B and C in DM group and non-DM group were 0.833 and 0.702, due to the sample size of 715, the power of test (1−β) exceeded 95% with α=0.05, according to the sample size calculation formula, The sample size was enough in our study. |
| Quantitative variables | 11 | Explain how quantitative variables were handled in the analyses. If applicable, describe which groupings were chosen and why  Continuous variables were described in mean and standard deviation (SD), and frequencies (%) were used for categorical variables. We used the Student t-test, Pearson Chi-squared test, analysis of variance (ANOVA) to compare the variables. The association between the number of occlusal supporting areas/Eichner index and DM was examined by logistic regression model. The number of occlusal supporting areas was treated as a continuous variable while the Eichner index as an ordinal categorical variable. |
| Statistical methods | 12 | (*a*) Describe all statistical methods, including those used to control for confounding  (*b*) Describe any methods used to examine subgroups and interactions  Continuous variables were described in mean and standard deviation (SD), and frequencies (%) were used for categorical variables. We used the Student t-test, Pearson Chi-squared test, analysis of variance (ANOVA) to compare the variables. The association between the number of occlusal supporting areas/Eichner index and DM was examined by logistic regression model. The number of occlusal supporting areas was treated as a continuous variable while the Eichner index as an ordinal categorical variable. The Odds ratio (OR) and 95% confidence intervals (CI) were presented as the measurement of the association. Model 1 was a univariate model and Model 2 and 3 were multivariable models. Model 2 was adjusted for age and sex; Model 3 was adjusted for confounders such as age, sex, body mass index, education, smoking and alcohol drinking, blood glucose and hypertension. The interaction between occlusal supporting areas/Eichner index and blood glucose was also included in model 3. In the model assessing the association between Eicher index and diabetes, group A was the reference group. |
|  |  |  |
|  |  | (*c*) Explain how missing data were addressed  No missing data. |
|  |  | (*d*) If applicable, explain how matching of cases and controls was addressed  Not applicable. |
|  |  | (*e*) Describe any sensitivity analyses  Not applicable. |
| Results | | |
| Participants | 13* | (a) Report numbers of individuals at each stage of study—eg numbers potentially eligible, examined for eligibility, confirmed eligible, included in the study, completing follow-up, and analysed |
|  |  | (b) Give reasons for non-participation at each stage |
|  |  | 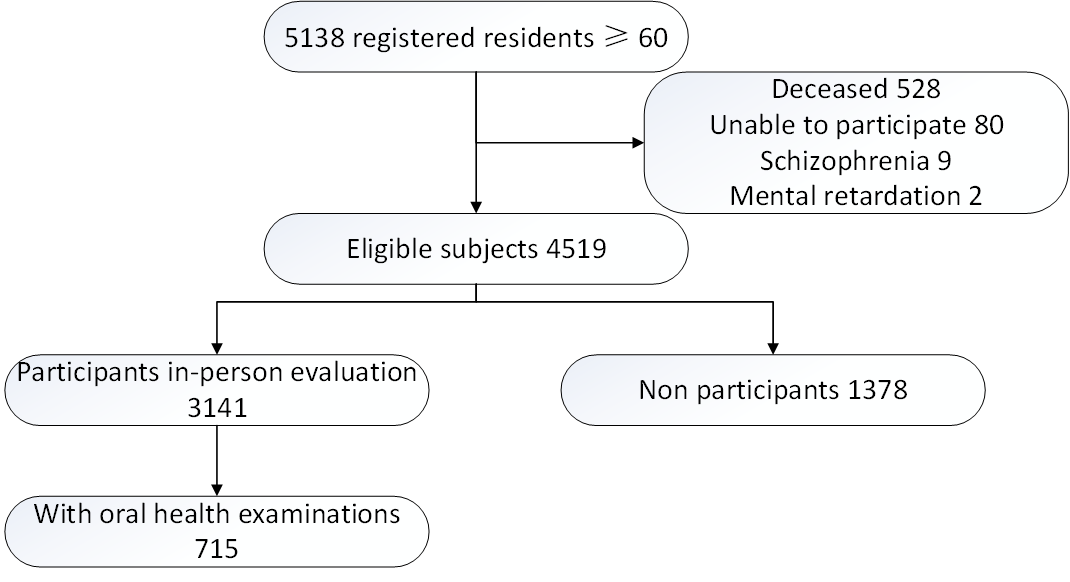(c) Consider use of a flow diagram |
| Descriptive data | 14* | (a) Give characteristics of study participants (eg demographic, clinical, social) and information on exposures and potential confounders  Refer to Table 1 |
|  |  | (b) Indicate number of participants with missing data for each variable of interest  No missing data. |
| Outcome data | 15* | Report numbers in each exposure category, or summary measures of exposure  Refer to Table 2 |
| Main results | 16 | (*a*) Give unadjusted estimates and, if applicable, confounder-adjusted estimates and their precision (eg, 95% confidence interval). Make clear which confounders were adjusted for and why they were included  Refer to Table 2 |
|  |  | (*b*) Report category boundaries when continuous variables were categorized  Not applicable. |
|  |  | (*c*) If relevant, consider translating estimates of relative risk into absolute risk for a meaningful time period  Not relevant. |

| Other analyses | 17 | Report other analyses done—eg analyses of subgroups and interactions, and sensitivity analyses  Not applicable. |
| --- | --- | --- |
| Discussion | | |
| Key results | 18 | Summarise key results with reference to study objectives  The average age of 715 participants was 73.74±6.49 years old. There were 84 diabetics with 1.71 occlusal supporting areas on average. Seven hundred and fifteen participants were divided into 3 groups according to Eichner classifications: Eichner group A with 4 occlusal functional areas, Eichner group B with 1-3 occlusal functional areas or 0 area with anterior occlusal contact, and Eichner group C with no functional occlusal contact. Blood glucose level was significantly lower in participants of Eichner group A compared to those in group B or C. The ordinal logistic regression showed more occlusal supporting areas were significantly associated with less DM cases with an Odds Ratio(OR) of 0.253(95%CI 0.108-0.594) after adjusting covariates. Participants in Eichner group C had a significantly much higher OR of 12.826 for DM (95%CI 1.44-114.214) compared to those in Eichner group A or B after adjustment. |
| Limitations | 19 | Discuss limitations of the study, taking into account sources of potential bias or imprecision. Discuss both direction and magnitude of any potential bias  Our study had several limitations. Firstly, the sample size might be relatively small. However, the sample size was proved to be enough after power analysis. Secondly, we did not measure the maximum bite force. As an indicator of masticatory performance, maximum bite force may also increase the risk of DM. However, the maximum bite force was closely related to functional occlusal area and Eichner index(Ikebe et al. 2005), and the effects of the latter two were analyzed respectively by using a multivariable logistic regression model to control confounding factors. Thirdly, the intake nutrients were so limited that some underlying nutrients might be neglected which caused metabolic disorders. Finally, the cross-sectional study was unable to reveal the causal relationship between occlusal support and DM. |
| Interpretation | 20 | Give a cautious overall interpretation of results considering objectives, limitations, multiplicity of analyses, results from similar studies, and other relevant evidence  In this study, the number of functional occlusal support areas may be inversely related to the blood glucose level and the prevalence of diabetes. There was no significant association between occlusal support and nutrients intake. We speculated that compared with poor mastication, good mastication might reduce postprandial blood glucose concentration by improving the digestion and absorption of nutrients. |
| Generalisability | 21 | Discuss the generalisability (external validity) of the study results  The participants were from Shanghai urban areas ,who was similar to those living in developed countries. Because other studies supported our findings, the results of the study could be generalized to those living in similar locations. |
| Other information | | |
| Funding | 22 | Give the source of funding and the role of the funders for the present study and, if applicable, for the original study on which the present article is based  Shanghai Municipal Science and Technology Major Project [2018SHZDZX01] and ZJ LAB, Key Project of the Ministry of Science and Technology, China [2021YFE0111800] supported data and sample collection. The Shanghai Stomatological Hospital School-level Key Department and Innovative Team Project [grant number SSDC-2019-ZDXK01,SSDC-2020-CXTD-A03] and Clinical Research Program from Shanghai Health Commission[grant number 2020YJZX0117,20194Y0142] supported analysis, interpretation of data and writing the manuscript. |

*Give information separately for cases and controls.

**Note:** An Explanation and Elaboration article discusses each checklist item and gives methodological background and published examples of transparent reporting. The STROBE checklist is best used in conjunction with this article (freely available on the Web sites of PLoS Medicine at http://www.plosmedicine.org/, Annals of Internal Medicine at http://www.annals.org/, and Epidemiology at http://www.epidem.com/). Information on the STROBE Initiative is available at http://www.strobe-statement.org.
